# The Isotropic Fractionator as a Tool for Quantitative Analysis in Central Nervous System Diseases

**DOI:** 10.3389/fncel.2016.00190

**Published:** 2016-08-05

**Authors:** Ivan E. Repetto, Riccardo Monti, Marta Tropiano, Simone Tomasi, Alessia Arbini, Carlos-Humberto Andrade-Moraes, Roberto Lent, Alessandro Vercelli

**Affiliations:** ^1^Neuroscience Institute Cavalieri Ottolenghi, Department of Neuroscience, University of TurinTurin, Italy; ^2^Child Study Center, Yale School of Medicine, New HavenCT, USA; ^3^Federal University of Rio de Janeiro Medical School, Macaé CampusRio de Janeiro, Brazil; ^4^Institute of Biomedical Sciences, Federal University of Rio de JaneiroRio de Janeiro, Brazil

**Keywords:** isotropic fractionator, cerebral ischemia, epilepsy, striatal lesion, neuroprotection

## Abstract

One major aim in quantitative and translational neuroscience is to achieve a precise and fast neuronal counting method to work on high throughput scale to obtain reliable results. Here, we tested the isotropic fractionator (IF) method for evaluating neuronal and non-neuronal cell loss in different models of central nervous system (CNS) pathologies. Sprague-Dawley rats underwent: (i) ischemic brain damage; (ii) intraperitoneal injection with kainic acid (KA) to induce epileptic seizures; and (iii) monolateral striatal injection with quinolinic acid (QA) mimicking human Huntington’s disease. All specimens were processed for IF method and cell loss assessed. Hippocampus from KA-treated rats and striatum from QA-treated rats were carefully dissected using a dissection microscope and a rat brain matrix. Ischemic rat brains slices were first processed for TTC staining and then for IF. In the ischemic group the cell loss corresponded to the neuronal loss suggesting that hypoxia primarily affects neurons. Combining IF with TTC staining we could correlate the volume of lesion to the neuronal loss; by IF, we could assess that neuronal loss also occurs contralaterally to the ischemic side. In the epileptic group we observed a reduction of neuronal cells in treated rats, but also evaluated the changes in the number of non-neuronal cells in response to the hippocampal damage. In the QA model, there was a robust reduction of neuronal cells on ipsilateral striatum. This neuronal cell loss was not related to a drastic change in the total number of cells, being overcome by the increase in non-neuronal cells, thus suggesting that excitotoxic damage in the striatum strongly activates inflammation and glial proliferation. We concluded that the IF method could represent a simple and reliable quantitative technique to evaluate the effects of experimental lesions mimicking human diseases, and to consider the neuroprotective/anti-inflammatory effects of different treatments in the whole brain and also in discrete regions of interest, with the potential to investigate non-neuronal alterations. Moreover, IF could be used in addition or in substitution to classical stereological techniques or TTC staining used so far, since it is fast, precise and easily combined with complex molecular analysis.

## Introduction

Since its introduction (Herculano-Houzel and Lent, 2005), the isotropic fractionator (IF) method represented a significant innovation in the field of quantitative neuroscience. The issue of counting neurons and other cell types in the nervous system has been fundamental in neuroscience since the past century. Generating accurate and reproducible quantitative estimates of both neuronal and non-neuronal populations is a crucial requirement in a number of experimental setups aiming at tracking developmental or neurodegenerative events in the nervous system, yet available protocols are hampered by a number of technical and procedural limitations.

A series of methods was developed in order to overcome the problem of overestimating cell number due to double counting of profiles in histological sections, because not all of the counted objects are contained exclusively in one section (Abercrombie, 1946). The same nucleus, in this case, could lie partly within one section, partly within the adjacent section(s). Therefore, the bigger the nuclear dimension is, in relation to the section thickness, the bigger the error could be.

A first attempt to overcome double counting was the Abercrombie’s correction factor (Abercrombie, 1946; Clarke, 1992), by which the new concept of nuclear-point came to light. A nuclear-point was for Abercrombie any geometrical point of the same relative position in all nuclei. Through this equation: *P* = *A*^∗^(*M*/*L* + *M*); where *P* is the average number of nuclear points, *A* is the gross number of nuclei seen in section, *M* the thickness of the section, and *L* the average length of nuclei, the authors assumed that the error would be negligible and estimated cell numbers more accurate. In the last decade of the previous century, stereology emerged as the method for cell counting. Stereology is a mathematical method that derives global quantities (i.e., volume, surface area and length, and also object number) from measurements obtainable on sections of a given structure (Weibel, 1981; Cruz-Orive, 1987). In this field, for many years, the Optical Fractionator method was considered the gold standard for counting neurons and even synapses. The Optical Fractionator method involves counting cells/neurons through optical dissectors in a uniform and systematic sample that constitutes a fraction of the region to be analyzed (Gundersen, 1986; West et al., 1991; West, 1999). Several software tools (e.g., StereoInvestigator, Neurolucida) were developed according to this method in order to assist researchers in quantitative analysis (Tomasi et al., 2011; Apolloni et al., 2014; Boido et al., 2014; Papageorgiou et al., 2014; Deidda et al., 2015; Dudok et al., 2015; Kawagishi et al., 2015). Nevertheless, the method remains complex and time-consuming, and some authors (Clarke, 1992) still recommend the Abercrombie’s correction factor when the profile is small compared to the section thickness.

Stereological methods such as the optical dissector and fractionator can estimate the number of cells and neurons in discrete brain regions (Korbo et al., 1990; Andersen et al., 1992). However, because these estimates are obtained from cell densities, the estimates precision is related to the homogeneity of neurons in the samples (West, 1999). Thus, these methods result inaccurate to quantify for example the total cell numbers in the brain. This could be done, however, but require the burden of dividing the brain into numerous regions of homogeneous cell density. Additionally, because stereological estimates are necessarily achieved by multiplying cell density by volume, the numbers obtained are not independent variables and therefore cannot be used in statistical comparisons against volume (Harrison et al., 2002).

The possibility to overcome methodological and time limitations of standard stereological cell counting techniques led neuroscientists to get new insights into evolutionary and developmental issues in different species (Collins et al., 2010; Gabi et al., 2010; Lent et al., 2012; Herculano-Houzel et al., 2015). Developed first by Herculano-Houzel et al. (2006) to work on high-scale measurements and comparative studies (Santiago et al., 2007; Azevedo et al., 2009, 2013), the IF method briefly consists in transforming highly anisotropic brain structures into homogeneous, isotropic suspensions of cell nuclei, which can be identified immunocytochemically as neuronal or non-neuronal nuclei, then counted. The method was recently applied also in pathological contexts, such as: age-related neuronal deficit in rats (Morterá and Herculano-Houzel, 2012); mouse model of autism (Chen et al., 2015); mouse model of Alzheimer’s disease (AD; Brautigam et al., 2012) and even on human specimens from AD patients (Andrade-Moraes et al., 2013). The IF method has been recently substantially improved by an automated machine for large-scale fractionation (Azevedo et al., 2013).

This potentially ground-breaking method has not yet been widely adopted mainly due to the lack in calibrating and/or validating works that compare the IF with standard counting techniques, except for the works of Bahney and von Bartheld (2014) on human and macaque monkey samples and Miller et al. (2014) in chimpanzee primary visual cortex. Because of its fast, precise and reliable cell/neuron counts, the IF could be a good strategy to investigate for example the neuroprotective effects of different compounds in different central nervous system (CNS) pathologies.

Here, we test the use of IF method in experimental models of CNS diseases, showing that it is as precise as stereological counts, but significantly faster. To our knowledge there are few papers attempting to apply this method to investigate the neuronal loss following (i) cerebral ischemia; (ii) epileptic seizures; and (iii) striatal lesion in rat models (i.e., Lopim et al., 2016 for epilepsy in rats).

Moreover, this procedure, in comparison to stereological counts, could be implemented and optimized to work down stream of a Fluorescence-Activated Cell Sorting (FACS) technique allowing further molecular investigations such as mic roarray RNA quantification or real time quantitative PCR. This possibility has been recently explored by other groups (Guez-Barber et al., 2012) by performing these molecular analyses on NeuN-sorted cells from a cell suspension of adult rat striata.

## Materials and Methods

### Animals

Two- to four-month-old Sprague-Dawley (SD) male rats (Harlan-Italy, San Pietro al Natisone, Italy) were used for producing the cerebral ischemia, epilepsy and striatal lesion models. All the experimental procedures involving live animals were performed in strict accordance with European Community Council guidelines 86/609/EEC (November 24, 1986), and with the Italian Ministry of Health and University of Turin institutional guidelines on animal welfare (law 116/92 on Care and Protection of living animals undergoing experimental or other scientific procedures; authorization number 17/2010-B, June 30, 2010). Additionally, an *ad hoc* Ethical Committee of the University of Turin approved this study. Animals were maintained with a 12:12 light/dark cycle. Food and water were provided *ad libitum* (standard mouse chow 4RF25-GLP, Mucedola srl, Settimo Milanese, Italy) Particular care was taken to minimize the number of animals, their discomfort and pain.

### Cerebral Ischemia

Permanent middle cerebral artery occlusion (MCAo) was performed according to Renolleau’s method (Renolleau et al., 1998). Briefly, SD rats (*N* = 3) were anesthetized with 5% isoflurane (Isoflurane-Vet 100%, Liquid, Merial Italy, Milan, Italy) vaporized in O_2_/N_2_O 30:70, and maintained at 1.5–2.5% isoflurane during surgery. The left MCA was exposed and electrocoagulated with a bipolar forceps (Jeweler 30665, GIMA, Milan, Italy). Then the ipsilateral common carotid artery (CCA) was clamped during 90 min to avoid collateral perfusion.

After 24 h the animals were sacrificed with an overdose of anesthetic, the brains were isolated and cut with a rat brain matrix in coronal slices at a thickness of 2 mm. Then the samples were processed for Triphenyl-Tetrazolium Chloride (TTC) staining. The TTC reaction was stopped substituting the TTC solution with 4% paraformaldehyde (PFA) in 0.1 M phosphate buffer (PB, pH 7.4) and the brain slices post-fixed for 2 weeks. In sequence, the two hemispheres of each slice were separated and processed with the IF method singly.

Control rats (*N* = 3) were sacrificed with an overdose of anesthetic, the brains were isolated, cut, and processed for TTC and IF method as above mentioned.

### Epileptic Seizures

Sprague-Dawley rats (*N* = 3) were intraperitoneally (i.p.) injected with 11 mg/kg kainic acid (KA; Tocris Bioscience, Bristol, UK) to induce epileptic seizures. One hour after the injection, rats showed the first symptoms (immobility, facial myoclonus, and head nodding), and only animals that reached the fourth and fifth stages of the Racine scale (Racine et al., 1972) were included in the study: stage 0, no seizures; stage 2, head nodding; stage 3, forelimb clonus; stage 4, rearing in addition to severe forelimb clonus; and stage 5, rearing and falling in addition to severe forelimb clonus. To minimize suffering and prevent mortality, 2 h following the symptoms onset a single i.p. injection of 4 mg/kg diazepam (Valium; Roche, Monza, Italy) blocked epileptic seizures within 30 min of administration. Control rats (*N* = 3) were i.p. injected with PBS.

Twenty-four hours after KA administration, the animals were sacrificed with an overdose of ketamine and perfused transcardially with 4% buffered PFA. Rat brains were isolated and post-fixed in 4% PFA for 2 weeks and the hippocampus from control and treated rats was carefully dissected and processed by the IF method.

### Striatal Lesion

The surgical protocol of quinolinic acid (QA) injection was performed according to the procedure described by Figueredo-Cardenas et al. (1994). SD rats (*N* = 3) were anesthetized with 5% isoflurane vaporized in O_2_/N_2_O 30:70, placed in a rat stereotaxic apparatus (Stoelting, Wood Dale, IL, USA) and maintained under 1.5–2.5% isoflurane during surgery. QA (210 nmol/2 μl; Sigma–Aldrich, St. Louis, MO, USA) was injected into the right striatum at the following coordinates: AP +0.6, MD +2.8, DV -5. The hemisphere contralateral to the QA injection served as control.

The animals were killed 30 days later with an overdose of ketamine and perfused transcardially with 4% buffered PFA. Dissected rat brains were post-fixed in 4% PFA for 2 weeks. The fixed brains were sliced in coronal sections with a rat brain matrix and the single striatum portions carefully dissected under a stereomicroscope with the help of a rat brain atlas (Paxinos and Watson, 2016). Striata from both sides were processed by the IF method.

### Isotropic Fractionator Method

The IF was performed as in Herculano-Houzel and Lent (2005). Briefly, after the neural tissue was properly fixed, small fragments were collected and placed into a glass tissue grinder, a saline-detergent solution (40 mM sodium citrate; 1% Triton^TM^ X-100, Sigma–Aldrich, St. Louis, MO, USA) was added, and through careful and constant translation and rotation movements of a tightly coupled pestle, the tissue was disrupted chemomechanically. This homogenization broke the cell but not the nuclear membrane. The nuclear suspension obtained after centrifugation and resuspension was stained with the fluorescent DNA dye 4′-6-diamino-2-phenylindole dihydrochloride (DAPI; DAPI, dilactate, D9564, Sigma–Aldrich, St. Louis, MO, USA). Aliquots from the isotropic suspension were charged into a hemocytometer (Neubauer chamber) and observed under fluorescence microscopy (Nikon Eclipse 80i). The average nuclei density was determined by counting the number of nuclei within sectors of the coverslipped hemocytometer (1 mm^2^ area; 0.1 mm depth) for four aliquots. The total number of cells originally present in the analyzed region was then obtained by multiplying density by the total volume of the suspension. To identify the fraction of neuronal nuclei among the total number of DAPI-stained nuclei, another aliquot of the isotropic suspension was collected and immunostained with mouse primary antibody against neuronal nuclear protein (NeuN, MAB377, Chemicon, Single Oak Drive, Temecula, CA, USA, 1:200 in PBS, overnight incubation at room temperature). Then, the samples were washed in saline and incubated at room temperature for at least 2 h with the secondary antibody (Cy3 conjugated anti-mouse donkey IgG, Chemicon, Single Oak Drive, Temecula, CA, USA; 1:200 in PBS) and normal donkey serum (1:10; D9663, Sigma–Aldrich, St. Louis, MO, USA). The percentage of neurons was obtained by counting the number of NeuN-labeled nuclei among at least 500 DAPI-stained nuclei. The non-neuronal cells were quantified as the difference between the total number of cells and the total number of neurons.

### Data Analysis

The values from each specimen were averaged and compared to achieve the *p*-value as follows: ischemia and striatal lesion specimens by paired Student’s *t*-test, two tails; epileptic seizure specimens by unpaired Student’s *t*-test, two tails. Data for Student’s *t*-test significance were performed in Microsoft Excel. Data were expressed as mean ± SEM (standard error of the mean) and differences were considered significant when *p* ≤ 0.05.

## Results

### Cerebral Ischemia

**FIGURE 1:**
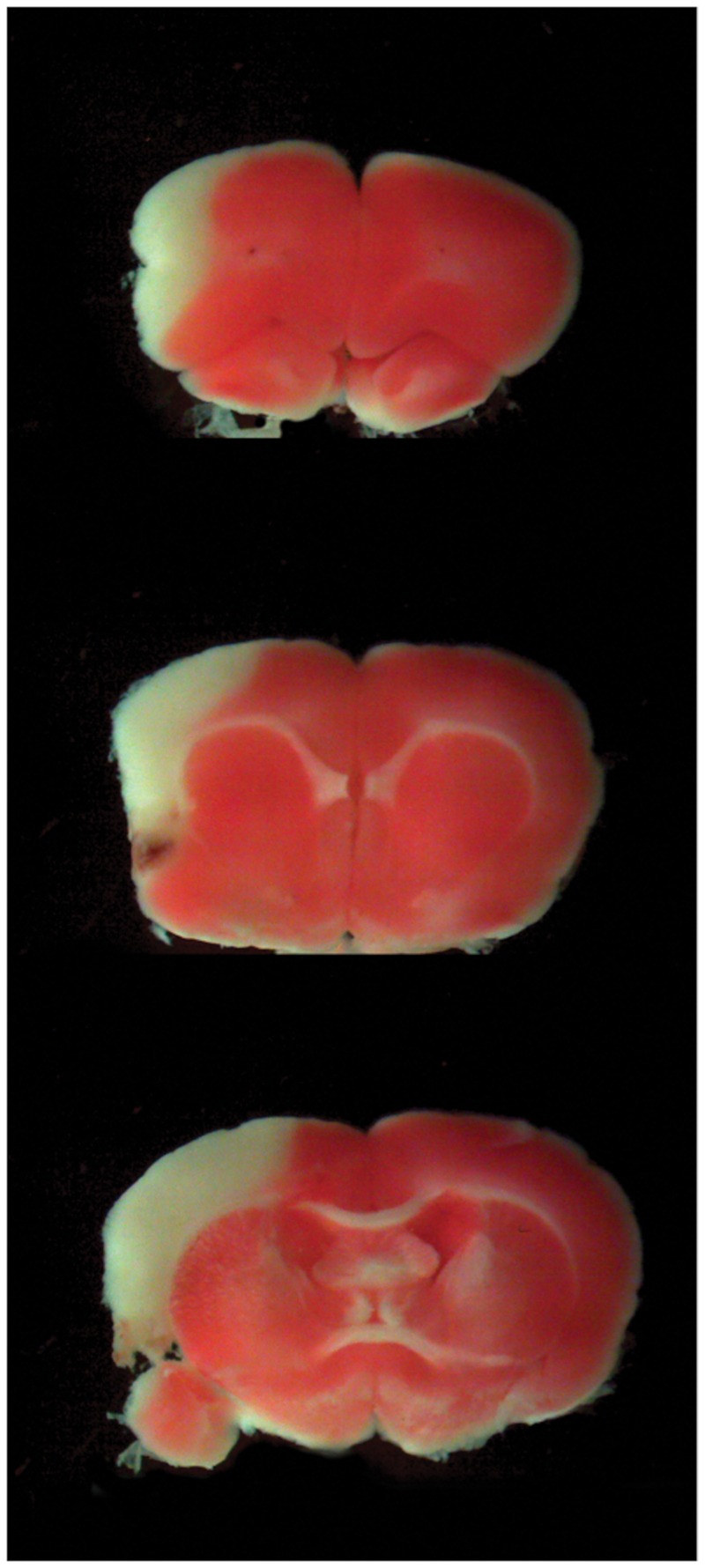
**Cerebral Ischemia.** TTC representative slices at different brain levels of the lesioned group. The percentage of ischemic volume was measured with Neurolucida software as described in the “Materials and Methods” section.

We first assessed the ischemic volume of the lesioned rats through TTC staining. Taking into account the formula for edema correction (Dohare et al., 2008), the ischemic lesion was 15.65% ± 0.44% of the whole brain, and the edema volume estimated to have an average value of 86.97 ± 20.43 mm^3^ affecting the 7.34 ± 1.58% of the whole brain (**Figure 1**).

We found a significant difference in the number of 4′-6-diamino-2-phenylindole dihydrochloride (DAPI) positive cells in ischemic brains compared to the controls (1.06^∗^10^8^± 3.95^∗^10^6^ vs. 1.33^∗^10^8^± 7.42^∗^10^6^, *p* = 0.04). Our cell counts in control brains are similar to cell/neuronal numbers observed in rats (Herculano-Houzel and Lent, 2005).

**FIGURE 2:**
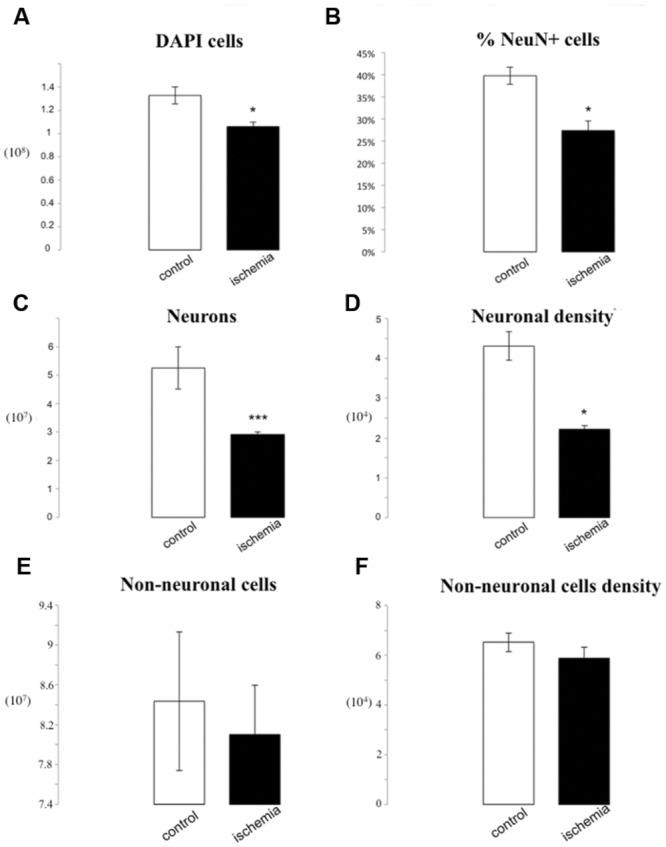
**IF quantification after cerebral ischemia.** Histograms representing the total number of DAPI positive cells **(A)**, the percentage of NeuN positive nuclei upon DAPI+ cells **(B)**, the total number of neurons **(C)**, and the neuron density, as cells/mg **(D)**, as well as the total number of non-neuronal cells **(E)** and non-neuronal density **(F)**. Data are presented as mean ± SEM and control vs. ischemic group (*T*-test, ^∗^*p* ≤ 0.5, ^∗∗∗^*p* ≤ 0.001, *n* = 3).

The percentage of NeuN positive cells was 27.44% ± 2.06 in lesioned rats as compared to 39.73% ± 1.94 in control rats (*p* = 0.01). That difference results in a 2.34^∗^10^7^ neuronal cell loss in ischemic brains as compared to the controls (2.91^∗^10^7^± 9.89^∗^10^5^ vs. 5.25^∗^10^7^± 8.75^∗^10^5^, *p* = 0.00006) and furthermore results in a significant reduction in neuronal density (2.22^∗^10^4^± 8.46^∗^10^2^ neurons/mg vs. 4.31^∗^10^4^± 3.59^∗^10^3^ neurons/mg, *p* = 0.02). There were no statistically significant differences in total number or density of non-neuronal cells between groups (7.70^∗^10^7^ ± 4.93^∗^10^6^ vs. 8.04^∗^10^7^ ± 6.95^∗^10^6^ *p* = 0.71; 5.88^∗^10^4^ ± 4.42^∗^10^3^ vs. 6.52^∗^10^4^ ± 3.75^∗^10^3^ non-neurons/mg *p* = 0.33; **Figure 2**).

**FIGURE 3:**
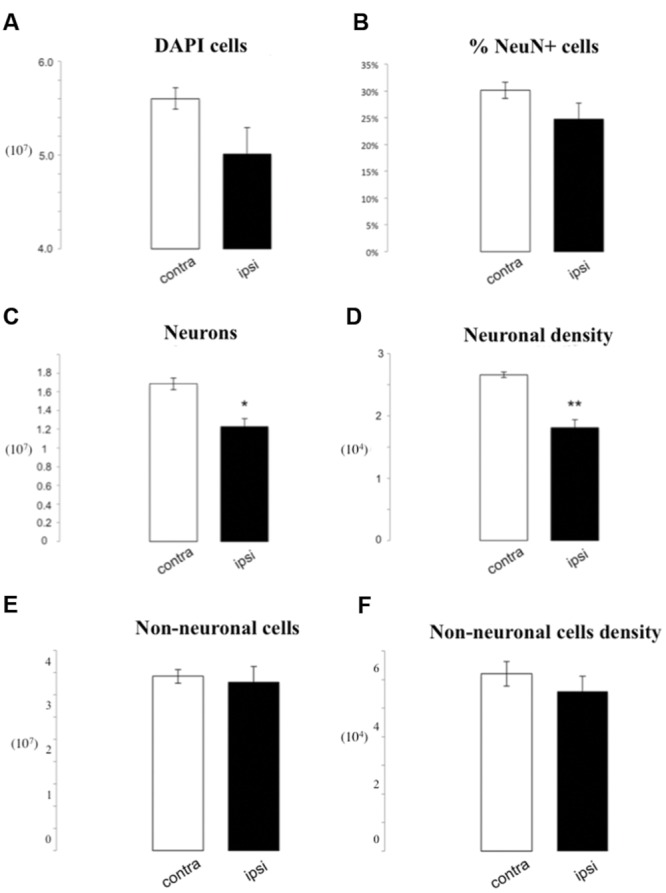
**IF quantification after cerebral ischemia, differences between ipsilateral and contralateral side after lesion.** Histograms representing the total number of DAPI positive cells **(A)**, the percentage of NeuN positive nuclei upon DAPI+ cells **(B)**, the total number of neurons **(C)**, and the neuron density, as cells/mg **(D)**, as well as the total number of non-neuronal cells **(E)** and non-neuronal cells density **(F)**. Data are presented as mean ± SEM and contra vs. ipsilateral side (*T*-test, ^∗^*p* ≤ 0.5, ^∗∗^*p* ≤ 0.01, *n* = 3).

We also analyzed in more detail the difference between ipsilateral and contralateral sides of lesioned brains. We observed a tendency for a decrease in total cell number loss on the side ipsilateral to the medial cerebral artery obstruction (MCAo), reaching 5.92^∗^10^6^ DAPI positive profiles reduction as compared to the contralateral side (5.01^∗^10^7^± 2.82^∗^10^6^ vs. 5.6^∗^10^7^± 1.14^∗^10^6^, *p* = 0.07). When we considered the percentage of NeuN positive nuclei, we found no differences between groups (24.74% ± 3.04 vs. 30.14% ± 1.51, *p* = 0.16). This corresponded to a depletion in the number of neuronal profiles after ischemia on the ipsilateral side (1.23^∗^10^7^± 8.45^∗^10^5^ vs. 1.69^∗^10^7^± 6.35^∗^10^5^, *p* = 0.05). Moreover, we found a statistically significant reduction in neuronal density (8.52^∗^10^3^ neurons/mg) on the ipsilateral side (1.81^∗^10^4^± 1.32^∗^10^3^ vs. 2.66^∗^10^4^± 4.32^∗^10^2^, *p* = 0.01). There were no statistically significant differences of the total number or density of non-neuronal cells between ipsilateral and contralateral sides (3.78^∗^10^7^ ± 3.47^∗^10^6^ vs. 3.92^∗^10^7^ ± 1.54^∗^10^6^ *p* = 0.74; 5.59^∗^10^4^ ± 5.29^∗^10^3^ vs. 6.20^∗^10^4^ ± 4.34^∗^10^3^ non-neurons/mg; *p* = 0.41; **Figure 3**).

### Epileptic Seizures

**FIGURE 4:**
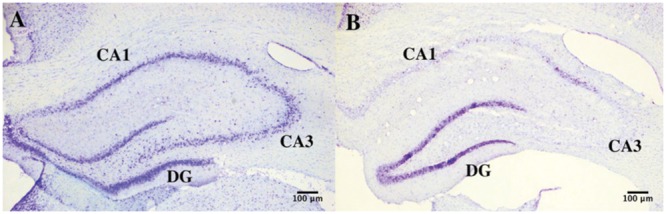
**Hippocampal degeneration.** Representative images of Nissl-stained sections of the hippocampus in control **(A)** and KA-treated **(B)** mice showing neuronal degeneration after KA injection.

**FIGURE 5:**
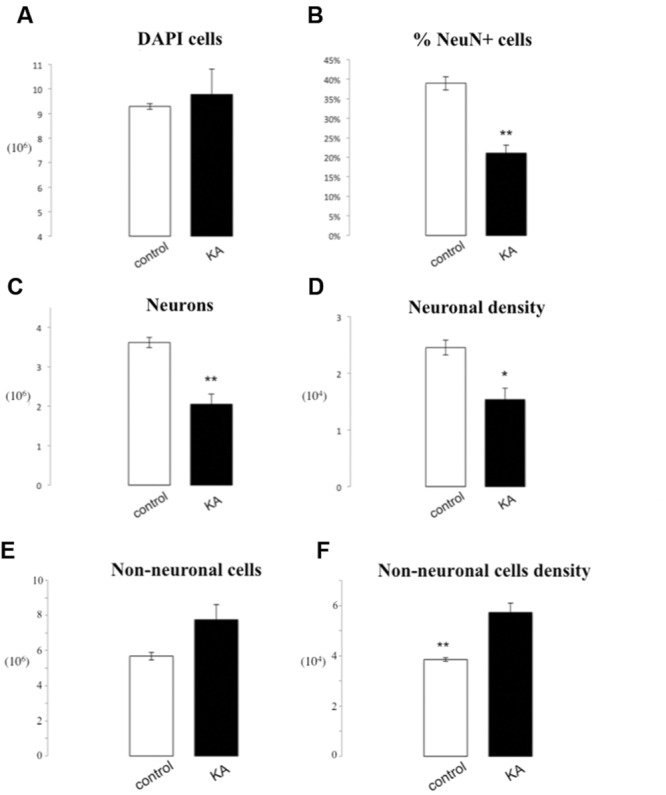
**IF quantification after KA administration.** Histograms representing the total number of DAPI positive cells **(A)** in the hippocampus, the percentage of NeuN positive nuclei upon DAPI+ cells **(B)**, the total number of neurons **(C)**, and the neuron density, as cells/mg **(D)**, as well as the total number of non-neuronal cells **(E)** and non-neuronal cells density **(F)**. Data are presented as mean ± SEM and control vs. ischemic group (*T*-test, ^∗^*p* ≤ 0.5, ^∗∗^*p* ≤ 0.01, *n* = 3).

The overall number of DAPI stained nuclei was unchanged after epileptic seizures induced by KA (9.29^∗^10^6^± 1.16^∗^10^5^ vs. 9.79^∗^10^6^± 1.03^∗^10^6^, *p* = 0.65). If we consider instead the number of NeuN+ nuclei, we observe a significant loss in the lesioned group following KA injection (2.04^∗^10^6^± 2.56^∗^10^5^ vs. 3.61^∗^10^6^± 1.26^∗^10^5^, *p* = 0.005). This caused a significant decrease of 46% of the number of neurons in the lesioned hippocampus (21.01% ± 2.1% vs. 38.91% ± 1.69%, *p* = 0.002). Also, neuronal density was significantly reduced (9.22^∗^10^3^ neurons/mg) following KA injection (1.53^∗^10^4^± 2.09^∗^10^3^ vs. 2.46^∗^10^4^± 1.30^∗^10^3^ *p* = 0.02). Concerning non-neuronal cells there was a tendency for an increase of the total non-neuronal cell number in lesioned rats as compared to controls (7.74^∗^10^6^ ± 8.68^∗^10^5^ vs. 5.68^∗^10^6^ ± 2.14^∗^10^5^ *p* = 0.08), while we observed a significant increase in non-neuronal cell density in lesioned hippocampus (5.73^∗^10^4^ ± 3.67^∗^10^3^ vs. 3.85^∗^10^4^ ± 7.31^∗^10^2^ non-neurons/mg *p* = 0.007; **Figures 4** and **5**).

### Striatal Lesion

**FIGURE 6:**
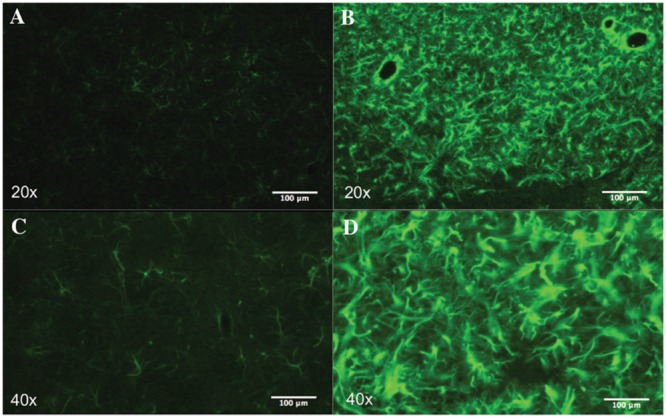
**Striatal astrogliosis.** Representative images of the striatum showing reactive astrogliosis after QA injection. Astrocytes were labeled with GFAP. In lesioned rats **(B,D)** there is a remarkable increase in GFAP immunoreactivity compared to the control group **(A,C)**.

**FIGURE 7:**
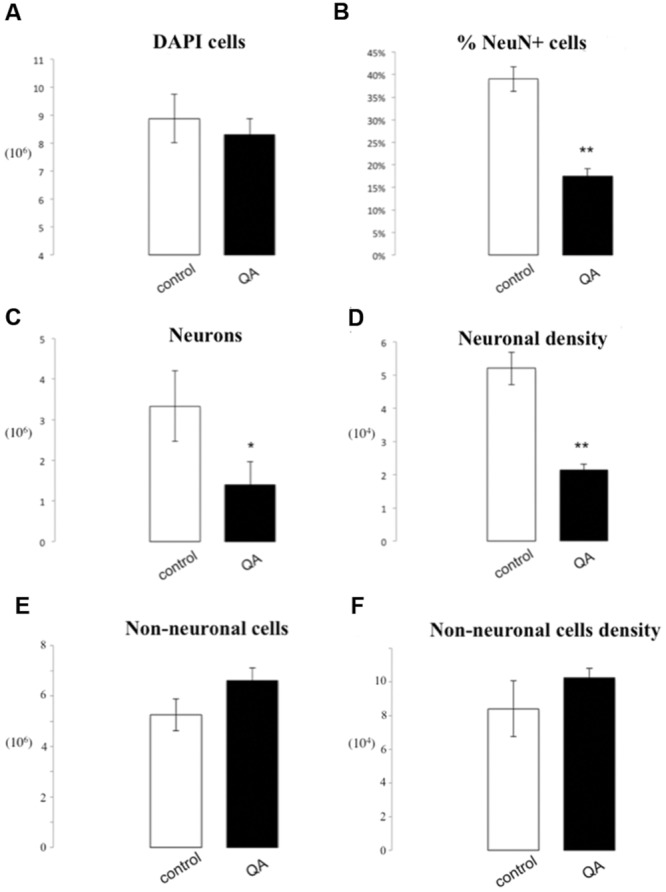
**IF quantification after QA injection.** Histograms representing the total number of DAPI positive cells **(A)**; the percentage of NeuN positive nuclei upon DAPI+ cells **(B)**, the total number of neurons **(C)**, and the neuron density, as cells/mg **(D)**, as well as the total number of non-neuronal cells **(E)** and non-neuronal cells density **(F)**. Data are presented as mean ± SEM and control vs. QA group (*T*-test, ^∗^*p* ≤ 0.05, ^∗∗^*p* ≤ 0.01, *n* = 3).

The number of DAPI positive nuclei in striata lesioned through parenchymal injection of QA was reduced by 5.81^∗^10^5^ (8.00^∗^10^6^± 5.67^∗^10^5^ vs. 8.59^∗^10^6^± 8.63^∗^10^5^, *p* = 0.76), a non-significant difference. Neuronal nuclei were significantly depleted in the QA-lesioned striatum (1.4^∗^10^6^± 1.46^∗^10^5^ vs. 3.33^∗^10^6^± 3.63^∗^10^5^, *p* = 0.04). This corresponded to a 21.5% reduction in the percentage of NeuN+ cells, statistically significant (17.5% ± 2.75% vs. 38.99% ± 4.7%, *p* = 0.003). Moreover, we observed a significant reduction of 3.04^∗^10^4^ neurons per mg in neuronal density of the QA lesioned striatum by (2.16^∗^10^4^± 1.54^∗^10^3^ vs. 5.2^∗^10^4^± 4.91^∗^10^3^, *p* = 0.02). There were no statistically significant differences for the number or density of non-neuronal cells in lesioned rats as compared to controls (6.61^∗^10^6^ ± 5.03^∗^10^5^ vs. 5.25^∗^10^6^ ± 6.25^∗^10^5^ *p* = 0.16; 1.02^∗^10^5^ ± 5.88^∗^10^3^ vs. 8.40^∗^10^4^ ± 1.66^∗^10^4^ non-neurons/mg, *p* = 0.35; **Figures 6** and **7**).

## Discussion

The aim of this study was to test the use of the IF method for the study of CNS diseases in order to provide a novel tool to assess quantitative parameters related to the effects of therapeutic strategies. The IF method was previously adopted for determining age-related neuronal loss in rats (Morterá and Herculano-Houzel, 2012), synucleinopathy (Aldrin-Kirk et al., 2014), in a mouse model of AD (Brautigam et al., 2012) and even on AD human patients (Andrade-Moraes et al., 2013). Here, we used it to detect changes in cell numbers occurring after cerebral ischemia, epileptic seizures and striatal lesion mimicking Huntington’s Disease (HD).

A major goal in the field of clinical neuroscience is to develop and characterize neuroprotective agents which could either reduce or delay brain damage, or modulate regenerative responses in the parenchyma (e.g., neurogenesis, angiogenesis, axonal sprouting, and synaptogenesis) which are directly stimulated by a lesion such as an ischemic insult (Zhang and Chopp, 2009).

In general, there are three major types of cell death induced by a pathogenic process: necrosis, apoptosis, and autophagic death. The characteristic features of apoptosis are cellular shrinkage and blebbing, nuclear fragmentation, and formation of apoptotic bodies (Kerr et al., 1972). Apoptosis is involved in neuronal cell loss in the penumbra of an ischemic brain injury (Mehta et al., 2007); in epileptic seizures (Cole-Edwards et al., 2006) and in Parkinson’s disease (PD; Ciechanover and Brundin, 2003; Olanow, 2007). Such a cellular death alters the morphology of cell nuclei and in turn the number of viable/not viable neurons that an automatic counting method for IF (Collins et al., 2010) can detect (i.e., counting a picnotic nucleus like a vial nucleus), causing a bias in particular in studies focused on neuronal cells rescue or replacement.

Noteworthy, preclinical research has a low translational success rate. Even if related to a specific pathology (e.g., stroke), the update of Stroke Therapy Academic Industry Roundtable (STAIR) recommendation pointed out (Fisher et al., 2009) that it is mandatory to improve sample size, rigor, standardization, and minimize bias within the experimental protocols. We think that especially for the first point, the IF is appealing for stroke studies because of its capability to obtain reliable results in short time over a large number of samples. Moreover, it would be useful not only for stroke research but also for the most of neuroprotection/neurorestorative studies upon different CNS diseases.

We believe that IF done by manual counting can give more consistent results than an automatic counting procedure based on FACS; nevertheless, within the flow-cytometry-based techniques for cell counting, the FAST-FIN could be the more promising especially for the capability to discriminate not only neuronal-vs. non-neuronal nuclei but also glial vs. neuronal cells (Marion-Poll et al., 2014), even if this characterization is made only on nuclear size.

Of great interest, especially for disease-related investigations, could be understanding whether a disease (such as stroke or epilepsy) could differentially affect different types of glial cells rather than neurons. There is a growing body of evidence supporting the notion that glial cells play a crucial role in pathogenesis and progression of CNS disorders (Takeuchi, 2013).

Unfortunately, even if a nuclear marker for oligodendrocytes is reliable and well characterized (the transcriptional factor SOX10, Kuhlbrodt et al., 1998) there are not so far appreciable nuclear markers for the other glial cell types that work upon this protocol. There are some unpublished data exploring the use of Olig2 in the IF method as a marker for specific type of oligodendrocytes in mouse models of psychiatric diseases, but the main issue to solve is whether it is universal (stains **all** oligodendrocytes in a brain region) and specific (stains **only** oligodendrocytes in that brain region). This issue has been solved for neuronal markers such as NeuN (Mullen et al., 1992; Wolf et al., 1996; Sarnat et al., 1998) but still not for those of glial subtypes.

### Cerebral Ischemia

In cerebral ischemia induced by a permanent MCAo in adult rats, we observed a significant reduction in the total number of nuclei in ischemic brains as compared to the controls. In fact, the decrease in the number of DAPI+ nuclei on ischemic brains (2.67^∗^10^7^) is very close to that of NeuN+ nuclei (2.34^∗^10^7^). During our examination we found that the percentage of NeuN+ nuclei in control brains was almost 40% of the total number of nuclei. This percentage is similar to that observed by Herculano-Houzel and Lent (2005). Slight differences may derive from different post-fixation times in the two papers. In fact, it is known that a longer fixation time could mask NeuN epitope retrieval (Gill et al., 2005). Also, since we used a different rat strain (SD instead of Wistar in Herculano-Houzel and Lent, 2005), it is possible that this could be a source of some difference in numbers. On the other hand, our neuronal counts are consistent with those from Aldrin-Kirk et al. (2014) in adult SD rat forebrains, especially regarding neuronal density (neurons/mg).

In the ischemic brains, we counted separately the ipsilateral side to the MCAo and the contralateral side. Interestingly, beside a significant reduction of 17.93% of NeuN stained cells in the ipsilateral side as compared to the contralateral, we found a significant difference also in the percentage of NeuN between the control hemispheres and contralateral side (39.73% ± 1.94% vs. 30.14% ± 1.51%, *p* = 0.019), suggesting an impact of the ischemic procedure beyond the directly affected hemisphere. In fact, these data are consistent with the well-known influence of a focal ischemia on the whole brain, causing neuronal damage also in the contralateral hemisphere through neuroinflammation and blood brain barrier (BBB) leakage (Garbuzova-Davis et al., 2013).

Therefore, we confirmed that neurons are more sensitive to a hypoxic insult than other cell types in the cerebral cortex. It is known that astrocytes, especially in the ischemic boundary zone (IBZ), may resist to a slight reduction in glucose and oxygen delivery, with a prolonged survival compared to neurons (Swanson et al., 2004; Zhao and Rempe, 2010). In addition, astrocytes are more resistant to oxygen and glucose deprivation *in vitro* (Panickar and Norenberg, 2005). Microglial cells seem to be resistant to an increase of Ca^2+^ influx into the cell membrane due to excitotoxicity reactive expression of the GluA2 subunit of α-Amino-3-hydroxy-5-methyl-4-isoxazole propionic acid (AMPA) receptors (Beppu et al., 2013).

An important advantage of our protocol employing the IF method in cerebral ischemia is highlighted by the percentage of NeuN positive cells between groups. Neuronal loss was found to be 44.54%, much higher than expected by an ischemic volume lesion assessed with TTC on 15.65% of the whole brain. Thus, IF was able to detect cell death out of the ischemic core, beyond the penumbra, reaching as far as the contralateral hemisphere. Even though apoptotic nuclei can be detected by stereological analysis even outside the ischemic core, its occurrence and quantification was hardly explored before probably due to the complex and time-consuming protocols.

Almost all recent works on neuroprotective/neurorestorative agents for cerebral ischemia use TTC staining (i.e., Yu et al., 2014; Zhu et al., 2014) or standard stereological techniques (i.e., Liu et al., 2014; Morris et al., 2014) in order to evaluate the ischemic damage. Even though all these analyses were followed by neurologic scores derived from different behavioral tasks, the use of IF to these methods could have brought more precise data on the outcome of treatment, with a reasonable time investment. With IF, any amelioration on behavioral tasks could be attributed to the number of neurons in extra core lesion areas, even in the contralateral hemisphere, which in turn could suggest whether an improvement is the result of physiological/induced plastic changes in the lesioned area/contralateral side (Takatsuru et al., 2013) or is a specific effect of the candidate neuroprotective agent administration.

### Epileptic Seizures

Kainic acid is an analog of glutamic acid that acts on both AMPA/kainate receptors. Experimentally, an i.p. KA injection is currently used to induce excitotoxic cell death (Yang et al., 1997; Wang et al., 2005; Chihara et al., 2009). In rodents, KA leads to recurrent seizures, behavioral changes, and subsequent degeneration of selective populations of neurons in the brain (McKhann et al., 2003; Tripathi et al., 2009; Spigolon et al., 2010; Zhao et al., 2012; Grande et al., 2014). Here, we observed that, in spite of a critical loss in neuronal nuclei (1.57^∗^10^6^), the overall number of nuclei is unchanged. This can be explained by the occurrence of massive reactive gliosis and astrocyte proliferation which mask neuronal loss. KA-induced neuronal death in fact activates microglia and astrocytes (Chen et al., 2005; Ravizza et al., 2005). In KA-induced hippocampal injury, microglial activation is believed to contribute to neuroinflammation and neurodegeneration, thus, a reduction in glial cells activation is followed by a reduction in neuronal cell death (Cho et al., 2008). Our findings suggest that the total number of nuclei counted by use of the IF is not changed. This could be due the increase in the number of non-neuronal nuclei (**Figure 5**). Astrocytes are the most prominent glial cell population in the CNS and a proliferative response of astrocytes to KA administration was already observed almost 35 years ago (Murabe et al., 1982). Recently, an increase in glial fibrillary acidic protein (GFAP) expression was shown from 1/3 days up to 1 month after KA intra-hippocampal injection (Bendotti et al., 2000). By contrast, GFAP expression in the hippocampus was not affected 1 day after KA systemic administration, while a 20% decrease was observed in the amygdala/pyriform cortex (Ding et al., 2000). However, our previous experiments showed a 20% increase of GFAP immunostaining 1 day after i.p. administration (Spigolon et al., 2010). The massive reduction of the number of hippocampal neuronal cells (43%) in KA treated group observed here with IF is similar to the one observed by our previous experiments with histological counts (40%, Spigolon et al., 2010). However, this neuronal reduction is different from the one observed by Lopim et al. (2016) with IF in Wistar rats (26% at 30 days post lesion), but it is important to point out that, rather then the rat strains, both the model (pilocarpine vs. KA) and the time points (1 day vs. 30 days) were different. From the translational point of view, the IF method applied to epileptic seizures could be used as a control for the number of glial cells that can be changed following anti-oxidant, anti-inflammatory/proliferative therapies (Chung and Han, 2003; Takemiya et al., 2006; Miyamoto et al., 2008; Gupta et al., 2009). Finally, regarding the histological type of neuronal death following epileptic seizures, sometimes the TUNEL staining for apoptotic death fails to show any significant increase (Spigolon et al., 2010) and the Fluoro-Jade B (FJB) staining used by other groups (Cole-Edwards et al., 2006) is not directly related to neuronal death. Even in this case, the IF could be helpful to rapidly control the KA-induced neuronal death, occurring by apoptosis rather than necrosis.

### Striatal Lesion

Huntington’s Disease is an autosomal dominantly inherited neurodegenerative disease, in which an expansion of the cytosine–adenine–guanine (CAG) repeat in the gene encoding for the N-terminal region of the huntingtin protein (htt) leads to the formation of a polyglutamine stretch (mhtt; Bano et al., 2011). The behavioral symptoms are typically involuntary choreiform movements, cognitive impairment, and mood disorders, eventually compromising daily functional abilities (Walker, 2007; Paulsen et al., 2008). Unilateral QA induced striatal lesions are highly reminiscent of histological (selective loss of GABAergic and cholinergic neurons) and neurochemical characteristics of HD in experimental animals (Delli Carri et al., 2013; Kumar et al., 2013; Pérez-Severiano et al., 2013; Serrano Sánchez et al., 2014). Overexcitation of *N*-methyl-D-aspartate (NMDA) receptors following QA administration results in: profound oxidative damage; lipid peroxidation; mitochondrial dysfunction; and apoptosis (Estrada Sánchez et al., 2008; Pérez-De La Cruz et al., 2012). In fact, we observed that the neuronal loss consists of 44.87% of NeuN+ nuclei after lesion. Furthermore, 30 days after QA injection we found a difference of 6.77% of DAPI+ nuclei. Also in this case, similarly to KA experiments on the hippocampus, the reduction in the number of neuronal nuclei is higher than the difference in the number of total nuclei. We can ascribe this finding to an important reactive gliosis, as suggested by an increase in the number of non-neuronal nuclei (**Figure 7**). Microglial activation in the pathogenesis of HD has been addressed by clinical studies demonstrating a direct correlation between abnormal microglial activity and disease progression (Tai et al., 2007). While microglial activation is unlikely to be the initiating event in these neurodegenerative diseases, it may cause cell death via various pathways. When activated, microglia produce cytotoxic substances including pro-inflammatory cytokines (e.g., TNF-α and IL-1β) and reactive oxygen species (e.g., hydrogen peroxide and superoxide). During acute inflammatory reactions there is also a rearrangement of the extracellular matrix, and matrix metalloproteinases (MMPs) involved in this process have a prominent role in microglial genesis (Kierdorf et al., 2013).

Besides, QA can alter the BBB (Guillemin, 2012), leaving the brain parenchyma permissive to the infiltration of inflammatory responsive cells. The importance of microglial activation was underlined, moreover, by an excellent paper by Neher et al. (2012), who found that inflamed microglia could phagocyte viable neurons. QA administration also leads to an intense astrogliosis (Björklund et al., 1986; Dihné et al., 2001).

All these mechanisms could contribute to explain our finding by a proliferation of glial-cells following excitotoxicity-induced neurodegeneration. Most studies on HD treatment use complex stereological counting techniques to assess the parenchymal damage in the striatum (Mazurová et al., 2014; Southwell et al., 2015). The IF method for analyzing a discrete region such as the corpus striatum could be an additional/substitute strategy to obtain lesion specific information of induced neuronal loss, and even an indirect quantification of reactive gliosis (when using anti-inflammatory/glial genesis compounds), by observing the difference among different cell populations between experimental groups.

In another interesting context, when injected into the striatum of adult rodents to model HD, QA strongly stimulates the subventricular zone (SVZ) and striatal neurogenesis (Tattersfield et al., 2004; Collin et al., 2005). An adult reactive neurogenic process was also obtained in zebrafish with a telencephalic administration of QA (Skaggs et al., 2014). Neural progenitor cells (NPCs) in the SVZ have been proposed as an endogenous source of new neurons that could be mobilized to repair brain circuits damaged due to injury or disease (Kernie and Parent, 2010). The high percentage (80%) of newborn neurons that succeed to differentiate (NeuN expression) 6 weeks after the lesion, as found by Collin et al. (2005), rises a question to our quantification analysis. To what extent our NeuN+ percentage is influenced by newly born neurons in the striatum vs. pre-existing neurons escaped from excitotoxicity-induced neuronal death?

Recently, Deierborg et al. (2009) labeled newborn cells by i.p. injection of bromo–deoxy–uridine (BrdU), or by green fluorescent protein (GFP)-expressing lentiviral vectors injected into the SVZ. They did not detect any GFP^+^ cells that co-labeled with NeuN into the lesioned striatum at any time point studied (1, 2, and 3 weeks post lesion; Deierborg et al., 2009). Despite evidence that a certain amount of reactive neurogenesis occurs after an ischemic insult in particular in the acute phase (Yamashita et al., 2006; Liu et al., 2009; Wei et al., 2011), the potential of the newborn cells to replace dying medium spiny neurons is controversial (Arvidsson et al., 2002; Luzzati et al., 2006). In fact, in the mouse model of slow progressive degeneration (*Creb1^Camkcre4^Crem^-/-^* double mutant mice) newborn neuronal cells show a transient existence and they do not express any specific marker of striatal projection neurons (Luzzati et al., 2011).

## Conclusion

Our results (summarized in Supplementary Table S1) support the use of IF as a simple and reliable method to evaluate the effects of experimental lesions mimicking human diseases and the outcome of therapeutic measures. Moreover, we have shown that IF can provide additional information about neuronal death and glial proliferation: the finding of new specific markers for detecting astroglial and microglial nuclei could further improve the method. The IF method in fact can miss some details when the cell loss is type- or even subtype-specific. Nevertheless, the IF allows to count quickly the amount of cell loss, and it can easily discriminate neurons and glia by NeuN IHC. In cerebral ischemia, this is a valid complement to the evaluation of the volume of the infarct, and also allows to detect cell loss in the surrounding penumbra and in the contralateral hemisphere avoiding time consuming stereological counts. This holds true also for more discrete structures, such as the hippocampus and the striatum, where the inhomogeneity of the areas and the tissue (with myelin fascicles intermingled to neurons), respectively, make stereological counts complicated.

## Author Contributions

IR contributed to the design of the work, performed acquisition, analysis and interpretation of all the experiments; drafted and revised the work, RM performed acquisition and analysis of most of the experiment and revised the work, MT performed part of the experiments (epileptic seizures) and revised the work, ST contributed to the conception and design of the work and revised it, AA performed part of the experiments and revised the work, C-HA-M contributed to the interpretation of the data and revised the work, RL contributed to the conception of the work and revised it AV contributed to the conception and design of the work and revised it.

## Conflict of Interest Statement

The authors declare that the research was conducted in the absence of any commercial or financial relationships that could be construed as a potential conflict of interest.
